# Effects of pitavastatin add-on therapy on chronic kidney disease with albuminuria and dyslipidemia

**DOI:** 10.1186/s12944-015-0164-5

**Published:** 2015-12-09

**Authors:** Masato Ohsawa, Kouichi Tamura, Hiromichi Wakui, Tomohiko Kanaoka, Kengo Azushima, Kazushi Uneda, Sona Haku, Ryu Kobayashi, Kohji Ohki, Kotaro Haruhara, Sho Kinguchi, Yoshiyuki Toya, Satoshi Umemura

**Affiliations:** Department of Medical Science and Cardiorenal Medicine, Yokohama City University Graduate School of Medicine, 3-9 Fukuura, Kanazawa-ku, Yokohama, 236-0004 Japan; Department of Nephrology, Yokohama Hodogaya Central Hospital, Yokohama, 240-8585 Japan

**Keywords:** Albuminuria, Chronic kidney disease, Diet therapy, Estimated glomerular filtration rate, Dyslipidemia, Pentosidine, Pitavastatin

## Abstract

**Background:**

In non-dialysis chronic kidney disease (CKD) patients with dyslipidemia, statin therapy is recommended to prevent cardiovascular complications. Dyslipidemia has been also shown to be an independent risk factor for the progression of CKD. However, it is still unclear whether statin therapy exerts an inhibitory effect on renal deterioration in CKD patients with dyslipidemia. The purpose of the present study was to examine possible therapeutic effects of statin add-on therapy on renal function as well as parameters of lipid and glucose metabolism, arterial stiffness and oxidative stress, in comparison to diet therapy, in CKD patients with dyslipidemia.

**Methods:**

This study was a randomized, open-label, and parallel-group trial consisted of a 12-months treatment period in non-dialysis CKD patients with alubuminuria and dyslipidemia. Twenty eight patients were randomly assigned either to receive diet counseling alone (diet therapy group) or diet counseling plus pitavastatin (diet-plus-statin therapy group), to achieve the LDL-cholesterol (LDL-C) target of <100 mg/dl.

**Results:**

The statin treatment by pitavastatin was well tolerated in all of the patients without any significant adverse events and the average dose of pitavastatin was 1.0 ± 0.0 mg daily after treatment. After the 12-months treatment period, LDL-C was significantly lower in the diet-plus-statin therapy group compared with the diet therapy group (diet vs diet-plus-statin: LDL-C, 126 ± 5 vs 83 ± 4 mg/dL, *P* < 0.001). On the other hand, the diet-plus-statin therapy did not significantly reduce albuminuria or delay the decline in eGFR compared with the diet therapy, and there was no relationship between the change in LDL-C and the change in eGFR or albuminuria. However, diet therapy as well as diet-plus-statin therapy exerted similar lowering effects on the pentosidine levels (diet therapy group, baseline vs 12 months: 40 ± 4 vs 24 ± 3 ng/mL, *P* = 0.001; diet-plus-statin therapy, 46 ± 7 vs 34 ± 6 ng/mL, *P* = 0.008). Furthermore, the results of multivariate regression analysis indicated that the change in pentosidine was a significant contributor to the change in eGFR (β = −0.536, *P* = 0.011).

**Conclusions:**

Although statin add-on therapy did not show additive renal protective effects, the diet therapy as well as the diet-plus-statin therapy could contribute to the reduction in plasma pentosidine in CKD patients with albuminuria and dyslipidemia.

## Background

The prevalence of chronic kidney disease (CKD) has been increasing all over the world, and CKD patients are frequently complicated with dyslipidemia. Dyslipidemia is reportedly associated with cardiovascular complications which are the most common cause of death in CKD patients, and statin is recommended for the treatment of dyslipidemia to prevent cardiovascular complications in non-dialysis CKD patients with dyslipidemia [1]. Dyslipidemia has been also shown to be an independent risk factor for the progression of CKD [2, 3]. In large observational studies, dyslipidemia is a risk factor for the initiation and progression of CKD [4, 5]. In addition, previous meta-analysis showed that statins for treatment of dyslipidemia may be beneficial for reduction of albuminuria and inhibition of renal deterioration in CKD patients [6, 7]. The results of basic research studies showed that renoprotective effects of statins would be, at least partly, due to the amelioration of inflammatory and fibrotic responses in the kidney via its inhibitory effects on oxidative stress, advanced glycation end product (AGE), monocyte chemoattractant protein-1 (MCP-1), and transforming growth factor-β (TGF-β) [8–11].

Therefore, these accumulated findings imply that a treatment for lipid abnormality might prevent the progression of CKD, and it is expected that statin therapy for treating dyslipidemia could slow the progression of renal deterioration. However, there is a lack of randomized controlled trials to directly compare the therapeuic efficacy of standard diet therapy and statin add-on therapy, or to determine the clinical target of serum low-density lipoprotein cholesterol (LDL-C) level for the prevention of CKD progression, particularly in CKD patients with mild dyslipidemia. Although a recent randomized controlled trial “The ASsessment of clinical Usefulness in CKD patients with Atorvastatin (ASUCA) study” is designed to determine whether statin exerts protective effects on renal function including estimated glomerular filtration rate (eGFR) and urine albumin-to-creatinine ratio (UACR) in CKD patients with dyslipidemia, the main results have not been reported yet [12]. In the present study, we examined possible therapeutic effects of statin add-on therapy on parameters of renal function such as eGFR and UACR, as well as parameters of lipid and glucose matabolism, oxidative stress and arterial stiffness, in comparison to diet therapy, in CKD patients with albuminuria and dyslipidemia.

## Methods

### Study design

This study was a randomized, open-label, and parallel-group trial comparing the therapeutic efficacy of diet therapy and diet-plus-statin therapy in non-dialysis CKD patients with albuminuria and dyslipidemia. It was conducted in accordance with the ethical principles of the Declaration of Helsinki and was approved by the ethics committees of Yokohama City University Hospital. The study was registered at the Clinical Trial Registry of University Hospital Information Network (UMIN Clinical Trials Registry: UMIN000002526; http://www.umin.ac.jp/ctr/).

### Study participants

CKD patients with dyslipidemia who had no history of treatment with lipid-lowering agents were eligible for the study if they were ≥20 years, with LDL-C ≥100 mg/dl and albuminuria catogories A2 or A3 (UACR ≥ 30 mg/g-creatinine). The estimated GFR (eGFR) was calculated using a revised equation for the Japanese population [13]. Exclusion criteria included CKD patients of G5 category (eGFR < 15 mL/min/1.73 m^2^) or on dialysis, uncontrolled type 1 or type 2 diabetes, hypothyroidism, and a history of CVD, and hypersensitivity to pitavastatin. All of the patients provided written informed consent before participation in the study, and follow-up was undertaken by each investigator.

### Study treatment

The eligible patients were randomly assigned either to receive diet counseling alone (diet therapy group) or diet counseling plus pitavastatin therapy (diet-plus-statin therapy group). In both therapy groups, patients were instructed to restrict the energy intake according to their pathological conditions and needs (25–30 kcal/kg-standard body weight/day), and take a diet including 25 % of energy from fat, <300 mg cholesterol daily and <25 g alcohol daily. Patients in diet-plus-statin therapy group were initially given 1 mg of pitavastatin once daily and the dose of pitavastatin was titrated up to 4 mg daily as needed during the 12 months active treatment period, to achieve the LDL-C level < 100 mg/dl. Laboratory measurements, clinic BP, and adverse events were estimated at baseline and after 6 and 12 months of treatment. Brachial-ankle pulse wave velocity (baPWV) was measured at baseline and after 12 months of treatment.

### Measurement of clinic BP and baPWV

The clinic BP was measured in a sitting position using a calibrated standard mercury sphygmomanometer and the recommended cuff size. Two measurements were taken at 1–2 min intervals, and their average was regarded as the clinic BP. The baPWV values were determined by a PP analyzer (model: BP-203-RPE2; Omron Healthcare, Kyoto, Japan), as described previously [14–16]. Pulse volume waveforms were recorded with sensors placed over the right brachial artery and both tibial arteries. The baPWV values obtained by this method are reportedly correlated with the aortic PWV determined by the catheter method [17].

### Laboratory measurement

Blood and urine sampling were performed in the morning after an overnight fast. Plasma pentosidine was determined using an ELISA kits (SRL laboratory, Tokyo, Japan). Other parameters were determined by routine methods in the Department of Clinical Chemistry, Yokohama City University School Hospital.

### Statistical analysis

Data were expressed as the mean ± SE or as a percentage. Significant differences between the therapy groups were assessed by unpaired *t*-test or nonparametric analysis using the Wilcoxon *U* test for variables that were not normally distributed. Differences between the therapy groups for categorical variables were analyzed using the Chi-square test. A repeated-measures ANOVA model was utilized continuous variables obtained during the 12 months of treatment. SPSS18.0 statistical software was used for statistical analysis. A value of *P* < 0.05 was considered statistically significant.

## Results

### Patient characteristics

Thirty one patients with CKD and dyslipidemia were screened for eligibility from September 2009 to January 2011. At the inclusion visit, two patients did not fulfill the selection criteria. One patient was lost to follow-up before randomization. The causes of CKD were chronic glomerulonephritis (*N* = 13), hypertensive nephrosclerosis (*N* = 12), diabetic nephropathy (*N* = 3) and other causes (*N* = 3). The eligible patients were randomly assigned to receive diet counseling alone (diet therapy group, *N* = 14) or diet counseling plus pitavastatin therapy (diet-plus-statin therapy group, *N* = 14). Table 1 shows the baseline characteristics of the participants. There was a significant difference in aspartate transaminase (AST) at baseline between the two therapy groups, and the other parameters in the baseline characteristics were similar in the two therapy groups. The anti-hypertensive and other medications are summarized in Table 2, and there were no significant differences between the diet therapy group and diet-plus-statin therapy group. The statin treatment by pitavastatin was well tolerated in all of the patients without any significant adverse events and the average dose of pitavastatin was 1.0 ± 0.0 mg daily after a period of 12 months of treatment.

**Table 1:** Demographic characteristics of the study groups at baseline

	Diet (*n* = 14)	Diet-plus-statin (*n* = 14)	*P* value
Age (years)	63.9 ± 3.3	60.6 ± 3.4	0.492
Gender (female/male)	4/10	4/10	0.661
Body mass Index (kg/m^2^)	24.4 ± 0.7	24.3 ± 0.9	0.934
Current smoking (n (%))	1 (7)	2 (14)	0.500
Alcohol (n (%))	4 (29)	3 (21)	0.500
Diabetes mellitus (n (%))	4 (29)	5 (36)	0.500
Hypertention (n (%))	12 (86)	12 (86)	0.702
Cerebrovascular disease (n (%))	0 (0)	1 (7)	0.500
Ischemic heart disease (n (%))	0 (0)	1 (7)	0.500
Cause of CKD (n (%)):			0.318
Diabetic nephropathy	1 (7)	2 (14)	
Chronic glomerulonephritis	6 (43)	7 (50)	
Nephrosclerosis	6 (43)	4 (29)	
Others	1 (7)	1 (7)	
Systolic BP (mmHg)	133 ± 4	127 ± 4	0.306
Diastolic BP (mmHg)	79 ± 2	76 ± 2	0.435
Heart rate (beats/min)	69 ± 3	74 ± 2	0.239
Plasma glucose (mg/dL)	113 ± 7	107 ± 4	0.614
HbA1c (%)	5.6 ± 0.1	5.6 ± 0.1	0.946
TC (mg/dL)	211 ± 6	207 ± 7	0.620
LDL-C (mg/dL)	139 ± 6	136 ± 6	0.797
HDL-C (mg/dL)	56 ± 3	56 ± 4	0.966
TG (mg/dL)	145 ± 14	154 ± 20	0.715
AST (U/L)	24 ± 1	20 ± 1	0.035
ALT (U/L)	22 ± 2	20 ± 3	0.265
Creatine kinase (U/L)	110 ± 13	106 ± 13	0.946
Serum creatinine (mg/dL)	1.2 ± 0.1	1.3 ± 0.1	0.593
Estimated GFR (mL/min/1.73 m^2^)	50.1 ± 4.9	48.6 ± 4.7	0.827
GFR category (n (%)):			0.500
G1	1 (7)	0 (0)	
G2	2 (14)	4 (29)	
G3a	5 (36)	3 (21)	
G3b	5 (36)	6 (43)	
G4	1 (7)	1 (7)	
G5	0 (0)	0 (0)	
UACR (mg/g-Cr)	1025 ± 465	801 ± 254	0.946
Albuminuria category (n (%)):			1.000
A2	7 (50)	7 (50)	
A3	7 (50)	7 (50)	

Values are means ± SE or number (percentage). *CKD* chronic kidney disease, *BP* blood pressure, *TC* total cholesterol, *LDL-C* low-density lipoprotein cholesterol, *HDL-C* high-density lipoprotein cholesterol, *TG* triglyceride, *AST* aspartate transaminase, *ALT* alanine transaminase, *GFR* glomerular filtration rate, *UACR* urine albumin-to-creatinine ratio

**Table 2:** Medication in the study groups at baseline

	Diet (*n* = 14)	Diet-plus-statin (*n* = 14)	*P* value
Antihypertensive agents (n (%))			
Angiotensin II receptor blockers	12 (86)	10 (71)	0.324
Angiotensin-converting enzyme inhibitors	2 (14)	0 (0)	0.241
Calcium-channel blockers	10 (71)	8 (57)	0.430
Diuretics	4 (29)	3 (21)	0.500
β-blockers	4 (29)	3 (21)	0.500
α-blockers	1 (7)	0 (0)	0.500
Central sympatholytic agents	0 (0)	1 (7)	0.500
Glucose-lowering agents (n (%))			
Insulin and insulin analougues	0 (0)	0 (0)	-
Sulfonylureas	0 (0)	2 (14)	0.241
α-glucosidase inhibitors	3 (21)	1 (7)	0.298
Biguanides	1 (7)	0 (0)	0.500
Thiazolidinediones	0 (0)	1 (7)	0.500
Lipid-lowering agents (n (%))	0 (0)	0 (0)	-
Antiplatelet agents (n (%))	2 (14)	3 (21)	0.500

Values are number (percentage)

### Lipid and glucose metabolism

Table 3 shows the parameters of lipid and glucose metabolism at baseline and after 6 and 12 months of treatment. There were no significant differences in the parameters of lipid metabolism including total cholesterol (TC), LDL-C, high-density lipoprotein cholesterol (HDL-C) and triglyceride between the two groups at baseline. After 12 months of treatment, TC was significantly reduced in the diet-plus-statin therapy group (baseline vs 12 months: TC, 207 ± 7 vs 157 ± 5 mg/dL, *P* < 0.001), but not in the diet therapy group (baseline vs 12 months: TC, 211 ± 6 vs 201 ± 6 mg/dL, *P* = 0.187; Table 3). With respect to the therapeutic efficacy to achieve the target LDL-C control after the treatment, while the reduction in LDL-C in the diet therapy group did not reach the statistical significance (baseline vs 12 months: LDL-C, 139 ± 6 vs 126 ± 5 mg/dL, *P* = 0.055; Table 3), LDL-C in the diet-plus-statin therapy group was significantly reduced so as to reach the LDL-C target of <100 mg/dl (baseline vs 12 months: LDL-C, 136 ± 6 vs 83 ± 4 mg/dL, *P* < 0.001; Table 3). Furthermore, although HDL-C and triglyceride were similar in the two groups after the treatment period (diet vs diet-plus-statin: HDL-C, 56 ± 4 vs 57 ± 5 mg/dL, *P* = 0.775; triglyceride, 149 ± 21 vs 122 ± 18 mg/dL, *P* = 0.252; Table 3), TC and LDL-C in the diet-plus-statin therapy group were significantly lower than those in the diet therapy group after the 12-months treatment period (diet vs diet-plus-statin: TC, 201 ± 6 vs 157 ± 5 mg/dL, *P* < 0.001; LDL-C, 126 ± 5 vs 83 ± 4 mg/dL, *P* < 0.001; Table 3). The parameters of glucose metabolism including fasting plasma glucose and HbA1C were comparable in the two therapy groups before and after the treatment period (Table 3).

**Table 3:** Comparison of the parameters of lipid and glucose metabolism, hepatic function and muscle damage in the diet therapy and diet-plus-statin groups

	Diet			Diet-plus-statin		
	Baseline	6 months	12 months	Baseline	6 months	12 months
Lipid metabolism:						
TC (mg/dL)	211 ± 6	220 ± 9	201 ± 6	207 ± 7	171 ± 5**†	157 ± 5**††
LDL-C (mg/dL)	139 ± 6	139 ± 7	126 ± 5	136 ± 6	94 ± 4**†	83 ± 4**††
HDL-C (mg/dL)	56 ± 3	57 ± 4	56 ± 4	56 ± 4	61 ± 5	57 ± 5
TG (mg/dL)	145 ± 14	198 ± 33	149 ± 21	154 ± 20	132 ± 19	122 ± 18
Glucose metabolism:						
Glucose (mg/dL)	113 ± 7	108 ± 4	115 ± 7	107 ± 4	108 ± 4	115 ± 9
HbA_1C_ (%)	5.6 ± 0.1	5.6 ± 0.2	5.5 ± 0.2	5.6 ± 0.1	5.7 ± 0.1	5.6 ± 0.2
Hepatic and muscle enzymes:						
AST (U/L)	24 ± 1	27 ± 2	27 ± 2	20 ± 1†	26 ± 5	22 ± 2
ALT (U/L)	22 ± 2	25 ± 2	24 ± 3	20 ± 3	29 ± 8	26 ± 6
CK (U/L)	110 ± 13	128 ± 20	161 ± 54	106 ± 13	131 ± 24	114 ± 16

Values are means ± SE. *TC* total cholesterol, *LDL-C* low-density lipoprotein cholesterol, *HDL-C* high-density lipoprotein cholesterol, *TG* triglyceride, *AST* aspartate transaminase, *ALT* alanine transaminase, *CK* creatine kinase. **P < 0.001 vs baseline; †*P* < 0.05, ††*P* < 0.001 vs diet therapy alone

In addition, the parameters of hepatic function including AST and alanine transaminase (ALT) and the parameter of muscle damage including creatine kinase (CK) did not increase after 6 and 12 months of treatment in the both groups, and there was no statistically significant difference between the two groups during the whole active treatment period (diet vs diet-plus-statin: AST, 27 ± 2 vs 22 ± 2 U/L, *P* = 0.290; ALT, 24 ± 3 vs 26 ± 6 U/L, *P* = 0.849; CK, 161 ± 54 vs 114 ± 16 U/L, *P* = 0.585; Table 3).

### Renal function and oxidative stress

Table 4 shows the parameters of renal function and oxidative stress at baseline and after 6 and 12 months of treatment. With respect to the parameters of renal function, eGFR and UACR were comparable in the diet-plus-statin and diet therapy groups at baseline (Table 4). After 12 months of treatment, eGFR showed similar decreases in the diet-plus-statin and diet therapy groups without significant differences between the two groups (diet vs diet-plus-statin: eGFR, 45.9 ± 4.3 vs 45.1 ± 5.3 mL/min/1.73 m^2^, *P* = 0.924; Table 4). In addition, diet-plus-statin therapy did not affect UACR and there were no significant differences in UACR between the two groups after 12 months of treatment (diet vs diet-plus-statin: UACR, 687 ± 249 vs 557 ± 186 mg/g-Cr, *P* = 0.779; Table 4). With respect to a maker of oxidative stress, pentosidine was significantly reduced in both groups after the treatment (diet therapy group, baseline vs 12 months: 40 ± 4 vs 24 ± 3 ng/mL, *P* = 0.001; diet-plus-statin therapy, 46 ± 7 vs 34 ± 6 ng/mL, *P* = 0.008), and there was no significant difference between the two groups (diet vs diet-plus-statin: 24 ± 3 vs 34 ± 6 ng/mL, *P* = 0.214; Table 4).

**Table 4:** Comparison of the parameters of renal function and oxidative stress in the diet therapy and diet-plus-statin groups

	Diet			Diet-plus-statin		
	Baseline	6 months	12 months	Baseline	6 months	12 months
Renal function:						
Estimated GFR (mL/min/1.73 m^2^)	50.1 ± 4.9	45.1 ± 4.3*	45.9 ± 4.3*	48.6 ± 4.7	45.6 ± 4.5*	45.1 ± 5.3*
UACR (mg/g-Cr)	1025 ± 465	671 ± 220	687 ± 249	801 ± 254	714 ± 233	557 ± 186
Oxidative stress:						
Pentosidine (ng/mL)	40 ± 4	28 ± 3*	24 ± 3*	46 ± 7	38 ± 7	34 ± 6*

Values are means ± SE. *GFR* glomerular filtration rate, *UACR* urine albumin-to-creatinine ratio. *P < 0.05 vs baseline

### Blood pressure and arterial stiffness

Clinic blood pressure (BP) and brachial-ankle pulse wave velocity (baPWV) are shown in Table 5. At baseline, there were no significant differences in the clinic BP and baPWV between the two groups. During the active treatment period, both clinic BP and baPWV did not exhibit significant changes in the diet-plus-statin therapy group as well as the diet therapy group (Table 5). Consequently, systolic and diastolic BP did not differ between the two groups after 12 months of treatment (diet vs diet-plus-statin: systolic BP, 130 ± 4 vs 130 ± 4 mmHg, *P* = 0.753; diastolic BP, 81 ± 2 vs 77 ± 2 mmHg, *P* = 0.413; Table 5). In addition, baPWV was comparable in the diet therapy group and diet-plus-statin therapy group after the treatment period (diet vs diet-plus-statin: baPWV, 1705 ± 87 vs 1833 ± 141 cm/sec, *P* = 0.218; Table 5).

**Table 5:** Comparison of clinic BP and arterial stiffness in the diet therapy and diet-plus-statin groups

	Diet			Diet-plus-statin		
	Baseline	6 months	12 months	Baseline	6 months	12 months
Clinic BP:						
Systolic BP (mmHg)	133 ± 4	131 ± 4	130 ± 4	127 ± 4	133 ± 5	130 ± 4
Diastolic BP (mmHg)	79 ± 2	81 ± 2	81 ± 2	76 ± 2	79 ± 3	77 ± 2
Arterial stiffness:						
baPWV (cm/s)	1661 ± 86	-	1705 ± 87	1919 ± 144	-	1833 ± 141

Values are means ± SE. *BP* blood pressure, *baPWV* brachial-ankle pulse wave velocity

### Factors associated with renal function

To examine possible factors related to renal functional regulation, we performed univariate and multivariate linear regression analyses. In univariate analysis, the change in eGFR was inversely correlated with the change in pentosidine (β = −0.580, *P* = 0.002; Table 6). However, there was no significant relationship between the change in eGFR and the change in LDL-C (β = 0.080, *P* = 0.684; Table 6). Furthermore, the results of multivariate linear regression analysis indicated that the change in pentosidine was a significant contributor to the change in eGFR (β = −0.536, *P* = 0.011; Table 6). On the other hands, the change in UACR was not correlated with the change in pentosidine or the change in LDL-C in both univariate and multivariate linear regression analyses (Table 7).

**Table 6:** Univariate and multivariate linear regression analyses of clinical factors affecting the change in eGFR

Variables	Univariate		Multivariate	
	β	*P*	β	*P*
Change in UACR	0.286	0.140	0.243	0.199
Change in pentosidine	−0.580	0.002	−0.536	0.011
Change in LDL-C	0.080	0.684	−0.125	0.543
Change in systolic BP	−0.256	0.189	−0.371	0.076
Age	0.093	0.636	0.196	0.365
Sex (male:1, female: 0)	0.176	0.370	0.183	0.384
BMI	0.148	0.451	0.052	0.776
			Model *R* ^2^ = 0.500	

*R* ^2^ = coefficient of determination. *eGER* estimated glomerular filtration rate, *UACR* urine albumin-to-creatinine ratio, *LD BP* blood pressure, *LDL-C* low-density lipoprotein cholesterol, *HDL-C*, high-density lipoprotein cholesterol

**Table 7:** Univariate and multivariate linear regression analyses of clinical factors affecting the change in UACR

Variables	Univariate		Multivariate	
	β	*P*	β	*P*
Change in eGFR	0.286	0.140	0.390	0.199
Change in pentosidine	−0.045	0.830	0.156	0.594
Change in LDL-C	0.216	0.271	0.239	0.354
Change in systolic BP	0.032	0.872	0.390	0.149
Age	0.091	0.645	−0.098	0.725
Sex (male:1, female:0)	−0.067	0.734	−0.262	0.323
BMI	0.101	0.610	0.090	0.697
			Model *R* ^2^ = 0.197	

*R* ^2^ = coefficient of determination. *UACR* urine albumin-to-creatinine ratio, *eGFR* estimated glomerular filtration rate, *LDL-C* low-density lipoprotein cholesterol, *BP* blood pressure

## Discussion

In the present study, although the diet-plus-statin therapy efficiently lowered LDL-C without an increase in adverse events in CKD patients with albuminuria and dyslipidemia, no significant additive beneficial effects on parameters of renal function (UACR, eGFR) were observed in the diet-plus-statin therapy compared to the diet therapy alone.

Concerning possible renal protective effects of statins, the results of previous studies including meta-analysis, which examined the benefits of statin therapy on renal function, are not consistent [7, 18, 19]. In a post-hoc analysis of “Management of Elevated Cholesterol in the Primary Prevention Group of Adult Japanese Study” (MEGA) study, pravastatin exerted a beneficial effect on proteinuria in patients with hypercholesterolaemia [20]. The result of other post hoc analysis of “Greek Atorvastatin and Coronary Heart Disease Evaluation” (GREACE) study showed that atorvastatin therapy prevented renal functional decline in untreated dyslipidemia patients [21]. On the other hand, it was reported that rosuvastain therapy increased proteinuria in the general population [22], and the results of a post-hoc analysis of “Prevention of Renal and Vascular End-stage Disease Intervention Trial” (PREVEND-IT) study showed that pravastatin did not exert beneficial effects on albuminuria and eGFR [23]. Moreover, in the recent two meta-analyses, lipid-lowering therapy with statin did not improve kidney outcomes [18, 19].

Therefore, in terms of the prevention of CKD progression, there is no direct clinical evidence identifying beneficial effects of statin therapy to inhibit or reverse CKD progression, and there are still insufficient data based on randomized clinical trial to recommend the target for acceptable LDL-C level in CKD patients. In line with this situation, in the present study, the diet-plus-statin therapy did not significantly reduce albuminuria or inhibit the decrease in eGFR compared with the diet therapy in CKD patients with dyslipidemia. In addition, univariate and multivariate linear regression analysis showed no significant correlation between change in LDL-C and change in eGFR or UACR.

A possible reason for the lack of renal protective effects by statin therapy in the present study may be due to characteristics of lipid metabolism of participants. Since the inclusion criteria for LDL-C level was LDL-C ≥100 mg/dl, baseline LDL-C levels in the diet and diet-plus-statin therapy groups were 139 ± 6 and 136 ± 6 mg/dL, respectively, thereby indicating that the study participants belonged to “mild” dyslipidemia. With respect to the contribution of baseline LDL-C level to the progression of renal injury, it has been reported that circulating LDL-C has a charge affinity for glycoaminoglycans in the glomerular basement membrane and cause a glomerulosclerosis and tubulointerstitial injury, indicating an important role of high LDL-C level in the progression of kidney desease defined as “lipid nephrotoxicity” [24, 25]. In fact, epidemiologic evidence indicates that greater baseline LDL-C levels are related to the more rapid progression of CKD [4, 5]. Therefore, it would be possible that the “mild “range of dyslipidemia at baseline in the participants masked the renoprotective effects by the statin thepary in the present study. Additionally, dietary changes by dietary therapy might affect these results. It has been reported that some nutraceuticals and functional foods such as nuts and fish oil could be beneficial for dyslipidemia and vascular function in situations where statins cannot be used [26].

As an important finding in the present study, diet therapy as well as diet-plus-statin therapy exerted similar lowering effects on the pentosidine levels in CKD patients with dyslipidemia. Furthermore, there was an inverse correlation between the change in pentosidine and the change in eGFR. These results suggest possible beneficial effects of diet therapy with or without statin therapy to suppress oxidative stress, with subsequent improving influence on the kidney injury in CKD patients with dyslipidemia.

The mechanisms responsible for the inverse correlation between the change in pentosidine and the change in eGFR may be due to improvements in systemic inflammatory state by the decease in pentosidine levels in CKD patients. Pentosidine is an AGE, formed by glycosylation and oxidation, that accumulates markedly in advanced stages of CKD [27]. Oxidative stress play a crucial role in the pathogenesis and progression of endothelial dysfunction, CKD and cardiovascular disease [28, 29]. Previous studies also showed a relationship between pentosidine and the markers of inflammation and monocyte activation [30]. In addition, plasma pentosidine was strongly correlated with the markers of inflammation, such as CRP, fibrinogen and IL-6 and the levels of soluble vascular cellular adhesion molecule-1 (sVCAM-1) which play an important role in the development of atherosclerosis [31, 32]. Thus, AGE, oxidative stress and proinflammatory cytokines may contribute to the systemic inflammation in CKD patients with dyslipidemia and accelerate the progression of CKD and atherosclerosis [27, 33–37].

To the best of our knowledge, this is the first report to show that diet therapy for dyslipidemia in CKD patients exerted a beneficial effect to reduce the plasma pentosidine level which could affect the CKD progression. AGE is known to be formed by a non-enzymatic reaction in diet [38], and dietary AGE have been considered to contribute to the increases in serum AGE concentration [39, 40]. With respect to the relationship between diet and pentosidine, diet-derived pentosidine is reportedly one possible origin of plasma pentosidine [39, 41]. However, possible effects of diet therapy for dyslipidemia on plasma pentosidine levels had never been examined to date. The results of the present study for the first time showed that the standard diet therapy with or without statin therapy could contribute to the reduction in plasma pentosidine, even without using specially prepared pentosidine-restricted diet, in non-dialysis CKD patients with albuminuria and dyslipidemia.

There were several limitations in this study. Firstly, this study was not a double-blinded placebo-controlled trial. It is difficult to assess the safety of diet therapy and diet-plus-statin therapy because of the absence of placebo groups. Secondly, the sample size of this study was small, and the population was heterogeneous for the cause of CKD including nondiabetic kidney diseases and diabetic kidney disease. Therefore, it should be emphasized that we cannot make any conclusive statement with respect to the relative efficacy of the statin add-on therapy for lipid lowering effects as well as renal and vascular protective effects, in comparison to diet therapy, in CKD patients with mild dyslipidemia. Definitely the present study should be classified as a pilot study for executing head-to-head clinical trials including the placebo group with increased number of participants of homogeneous population in the background of CKD in the near future. Thirdly, we did not investigate the actual totality of dietary changes in participants between before and after the interventions, although we set the dietary goal. This limitation might make it difficult to evaluate the statin add-on effect exactly. Additionally, since diet therapy may exert several effects on glucose metabolism, adipose tissue function and other systems in addition to lipid metabolism, it is needed to examine the effects of diet therapy on other parameters such as insulin resistance, adipokines and endothelial function in further studies.

## Conclusions

In summary, the diet-plus-statin therapy did not exert significantly additive beneficial effects on parameters of renal function (UACR, eGFR), compared to the diet therapy, in spite of efficient LDL-C lowering by the diet-plus-statin therapy without an increase in adverse events in CKD patients with albuminuria and dyslipidemia. However, diet therapy as well as diet-plus-statin therapy exerted similar lowering effects on the pentosidine levels in these patients. Furthermore, the change in plasma pentosidine was inversely correlated with the change in eGFR. These results suggest that diet therapy as well as statin therapy are important as multifactorial treatment strategy for non-dialysis CKD patients with albuminuria and dyslipidemia.
